# Visit-to-visit glycemic variability is a strong predictor of chronic obstructive pulmonary disease in patients with type 2 diabetes mellitus: Competing risk analysis using a national cohort from the Taiwan diabetes study

**DOI:** 10.1371/journal.pone.0177184

**Published:** 2017-05-10

**Authors:** Hsien-Tsai Chiu, Tsai-Chung Li, Chia-Ing Li, Chiu-Shong Liu, Wen-Yuan Lin, Cheng-Chieh Lin

**Affiliations:** 1 Department of Public Health, College of Public Health, China Medical University, Taichung, Taiwan; 2 Clinical Research Outcome and Training Center, Big Data Center, China Medical University Hospital, Taichung, Taiwan; 3 Graduate Institute of Biostatistics, College of Public Health, China Medical University, Taichung, Taiwan; 4 Department of Healthcare Administration, College of Medical and Health Science, Asia University, Taichung, Taiwan; 5 School of Medicine, College of Medicine, China Medical University, Taichung, Taiwan; 6 Department of Medical Research, China Medical University Hospital, Taichung, Taiwan; 7 Department of Family Medicine, China Medical University Hospital, Taichung, Taiwan; National Yang-Ming University, TAIWAN

## Abstract

**Background:**

This study aims to examine the association between visit-to-visit glucose variability, which was measured by coefficient of variation (CV) of fasting plasma glucose (FPG) and hemoglobin A1c (HbA1c), and risk of chronic obstructive pulmonary disease (COPD) in a large number of patients with type 2 diabetes with an average follow-up of 7.58 years.

**Methods:**

We conducted a retrospective cohort study on 27,257 patients with type 2 diabetes who participated in the National Diabetes Case Management Program in Taiwan. Visit-to-visit variability in HbA1c and FPG at baseline and the incidence of COPD were analyzed using a modified Cox proportional hazards model considering competing risks.

**Results:**

A total of 2,346 incident cases of COPD. Patients were grouped into tertiles of FPG-CV and HbA1c-CV. The incidence rates in the first, second, and third tertiles were 9.87, 11.06, and 13.19, respectively, for FPG-CV and 10.2, 11.81, and 12.07, for HbA1c-CV per 1000 person-years. After adjusting for age, gender, diabetes duration, treatment type, smoking, hypertension, hyperlipidemia, baseline FPG and HbA1c levels, and complications, both FPG-CV and HbA1c-CV were independently associated with COPD. The hazard ratios of COPD for the third terile compared with the first tertile of FPG-CV were 1.26 (95% confidence interval [CI]: 1.13–1.40). Moreover, the hazard ratios of COPD for the third and second tertiles compared with the first tertile of HbA1c-CV were 1.13 (1.02–1.25) and 1.13 (1.02–1.26), respectively.

**Conclusions:**

Patients with FPG-CV higher than 34.6% or HbA1c-CV higher than 8.4% exhibited an increased risk of COPD. This finding confirmed the linear relationship of FPG-CV and HbA1c-CV to COPD. Visit-to-visit variability in FPG and HbA1c levels are strong predictors of COPD in patients with type 2 diabetes. Future studies should focus on lung dysfunction in diabetes, and adequate glucose control strategy in regular clinical practices must be established for COPD prevention.

## Introduction

Diabetes mellitus (DM), a group of chronic disorders, poses significant public health concern. The global incidence of diabetes increased from approximately 176 to 410 million from 1990 to 2013, and the rank of diabetes progressed from the 10th to the 7th top cause of global years lived with disability (YLD) [1]. According to the International Diabetes Federation, diabetes caused 4.9 million deaths and 612 billion US dollars worldwide in healthcare expenditure in 2014. The prevalence of diabetes persistently increases in all countries, projecting global cases estimated up to 592 million by 2035 [2]. In addition, type 2 diabetes became a major public health challenge in Asia, including Taiwan [3]. In the past decade, the prevalence and incidence rates of diabetes in Taiwan increased by over 50% and 10%, respectively [4, 5]. This chronic illness is not only an imperative medical or health issue because of the rapid increase in diabetes incidence worldwide but also an emerging social or financial problem that must be addressed by governments.

Fasting plasma glucose (FPG) and plasma glycated hemoglobin (HbA1c) levels are common measures used for diabetes diagnosis and assessment of glycemic control in clinical practice for diabetes measurement [6]. FPG indicates plasma glucose levels after fasting for 8–12 hours [7] and is often used in clinical practice for high accessibility and economic feasibility to obtain immediate information. HbA1c represents long-term glycemic control by detecting the states of sustained hyperglycemia in the proceeding 2 to 3 months [8]. Compared with FPG, HbA1c is the current gold standard for monitoring blood glucose control and has a well-established association with diabetes complication risk [9–11]. Nevertheless, HbA1c provides incomplete information with regard to glycemic variability (GV), which contributes to diabetes-related complications [12]. In addition to single FPG or HbA1c measurement, GV is an emerging marker of blood glucose control and the target for diabetes management; increasing lines of evidence suggest that oscillating glucose levels could be an independent risk factor associated with cardiovascular diseases (CVD) and other diabetes complications [13–15]. Hence, GV is a critical component of glycemia, which cannot be characterized by conventional measures of glycemic control, such as FPG and HbA1c levels [16].

Diabetes is associated with abnormalities in markers of systemic inflammation and increased risk of other diseases. Previous studies found a correlation between diabetes and impaired lung function [17–20]. A cross-sectional study on approximately 4,000 elderly females found that the measures of lung function were inversely correlated with the presence of insulin resistance and type 2 DM [21]. Moreover, hyperglycemia has been associated with poor outcomes in hospitalizations caused by exacerbation of chronic obstructive pulmonary disease (COPD), resulting in prolonged hospital stays and premature death [22]. Another study on patients with type 2 diabetes revealed that extreme plasma HbA1 values (indicative of hypoglycemia or hyperglycemia) are associated with increased risk of COPD [23]. Researchers postulated that GV plays a vital role in the pathogenesis of diabetes-related complications by increasing oxidative stress and endothelial dysfunction [24, 25]; however, the relationship between GV and COPD has not been determined yet. To our best knowledge, no previous studies investigated GV and the incidence of COPD in patients with type 2 diabetes. Supportive data are thus needed to elucidate the effect of oscillating blood glucose on COPD. Hence, in the present study, we analyzed the relationship between the extent of GV and the risk of COPD in Chinese patients with type 2 diabetes, who participated in the National Diabetes Case Management Program (NDCMP) in Taiwan.

## Methods

### Study population

**Fig 1:**
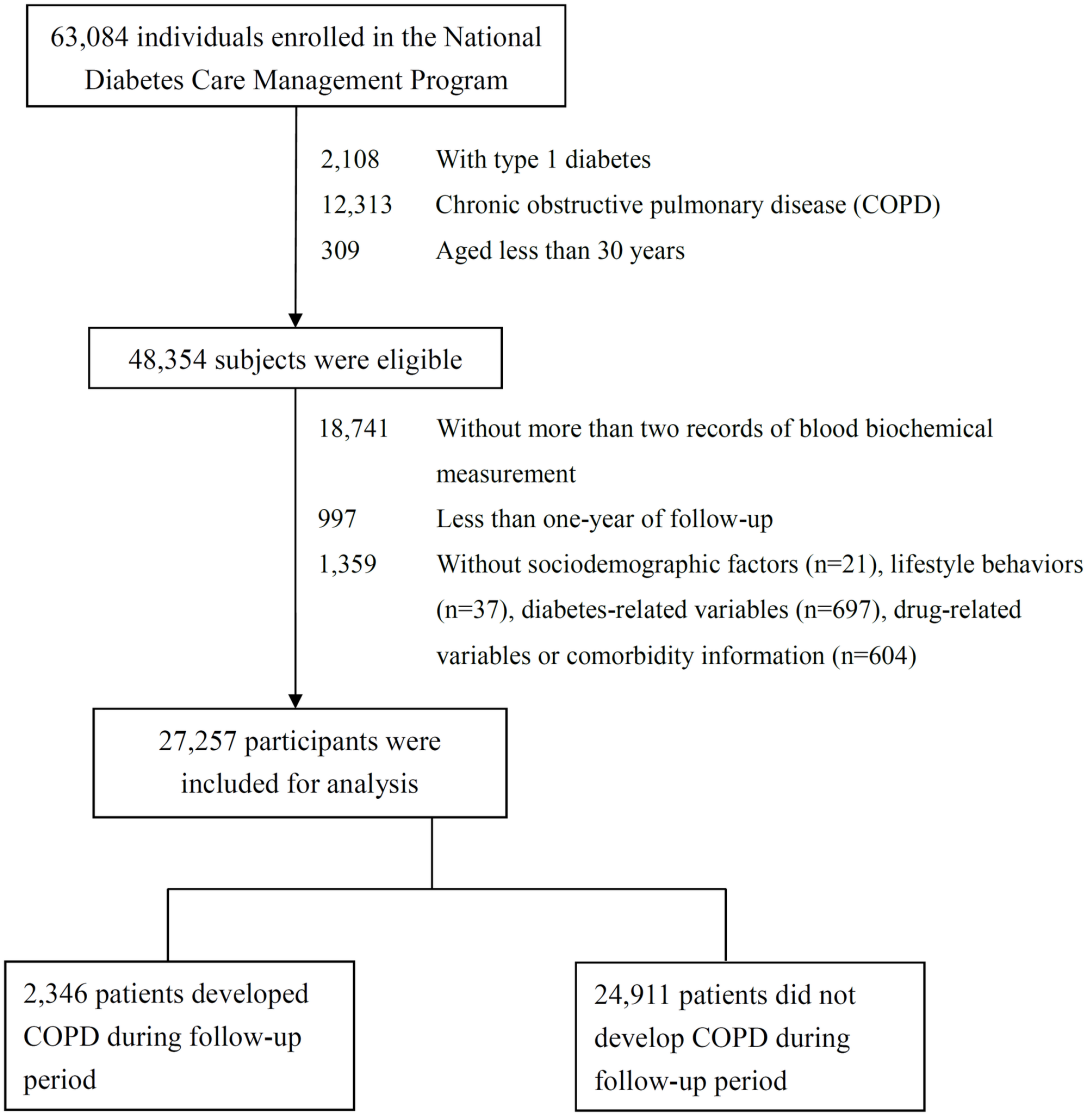
Flowchart of recruitment procedures for the current study.

We conducted a population-based cohort study, named the Taiwan Diabetes Cohort Study. The cohort consists of 63,084 ethnic Chinese patients with type 2 diabetes enrolled in the NDCMP in Taiwan during 2002–2004. NDCMP is a case management program established by the National Health Insurance (NHI) Bureau of Taiwan in 2002. We used the date of entry to the NDCMP as the index date. Clinically confirmed DM cases determined according to the American Diabetes Association criteria (International Classification of Disease, Ninth Revision, Clinical Modification [ICD-9-CM] diagnosis code 250) were eligible to enroll in this program. Exclusion criteria were as follows: type 1 diabetes (ICD-9-CM code 250.x1/x3), gestational diabetes (ICD-9-CM code 648.83), COPD at baseline, and aged 30 years or less. All eligible patients providing at least 1-year duration of follow-up for assessing COPD incidence and at least two biochemical measurements were included in the study. These inclusion criteria were met by 30,847 consecutively enrolled diabetic patients. A total of 27,257 patients were included in the data analysis after excluding those with missing data for sociodemographic factors, lifestyle behavior, diabetic complications, diabetes-related variables, drug-related variables, or comorbidity information (Fig 1). The distribution of baseline sociodemographic factors, lifestyles, diabetes-related variables, drug-related variables, comorbidity and blood biochemical measurement were comparable between patients included and excluded (S2 Table). The current study was approved by the Ethical Review Board of the China Medical University Hospital (CMUH102-REC3-016).

### Data sources for baseline and follow-up assessments

In March 1995, the Taiwanese government launched the NHI program. This insurance program has covered approximately 99% of the 23.74 million people in Taiwan since 1999. By the end of 2010, the NHI program covered more than 99.62% of the Taiwanese population, and the NHI Bureau established contracts with 100% of hospitals and 92% of clinics all over the nation. The insurance system audited claims data randomly. A random sample for every 50 to 100 outpatient and inpatient claims in every hospital and clinic are reviewed by expert committees quarterly for assuring the validity of the claims data. The NHI Bureau gives a severe penalty on every false diagnostic report. All information of health care utilization of NDCMP enrollees has been covered by the National Health Insurance Research Database (NHIRD) because the NDCMP was initiated by the NHI Bureau. In the current study, we analyzed 10-year (2002–2011) datasets of inpatient care for admissions and outpatient care visits. A unique personal identification number (PIN) is given to every individual in Taiwan. All data concerning patient identities are cryptographically scrambled by the NHIRD for assuring security and protecting privacy. Linkage among all NHI datasets can be achieved with the use of individual’s scrambled PIN. The contents of NHI datasets include information on basic demographic characteristics, ambulatory care, inpatient admission, date and source of diagnosis, and treatments in either outpatient or inpatient practices for all insured population. The ICD-9-CM is used to determine the health status of individuals. The proportion of participants’ withdrawal from the program is considerably low because of its comprehensive coverage. Therefore, the bias arising from loss to follow-up is trivial.

The NDCMP is a program conducted by the Ministry of Health and Welfare of Taiwan since 2001. This program mainly aims to enhance the quality of diabetes care by augmenting the frequency of monitoring, improving continuity of care, and preventing diabetes-related complications. Diabetes care providers, comprising physicians, diabetologists, dietitians, and diabetes educators, are involved in the program to share information and provide medical services according to their expertise. These diabetes care providers must be validated and certified by a diabetes training course. All enrollees are required to have 3 monthly physician visits, which include drug adjustment, diabetes education, and self-care and nutrition consultation. Retinal and peripheral neuropathy examinations are performed annually. When entering the NDCMP, all patients took a comprehensive assessment of their disease and complication status as well as a series of tests from blood and urine samples, and body measurements. Besides, enrollees are administered a standardized and computerized questionnaire by a case management nurse to record previous or current status of their lifestyle behavior, disease, and medication. NDCMP provides education including encouragement to exercise regularly and frequently self-monitor blood sugar levels. The program also includes modification of the lifestyle behavior of patients and provision of information for foot care, subcutaneous insulin injection techniques, and nutrition intake. Blood samples are collected from the antecubital vein in the morning, after fasting 12 h overnight, and then sent for analysis within 4 h after collection.

### Outcome ascertainment

COPD incidence was investigated from claim data of inpatient and outpatient based on major diagnosis codes from the ICD-9 (ICD-9-M codes 490–496). The NHIRD database were searched for this population to identify incident COPD patients with at least one inpatient claim or at least three outpatient claims. By linking the unique identification number with the computerized file, a total of 2346 patients suffering from COPD were identified within 8 years of follow-up from this cohort. The cohort was followed up from the index date to December 31, 2011 or until a COPD event, withdrawal from the NHI program, or death. Data on other chronic medical conditions were retrieved for a 1-year period before cohort entry by using outpatient and inpatient claim data. The histories of cancer (ICD-9-CM codes 140–149, 150–159, 160–165, 170–175, 179–189, 190–199, 200, 202, 203, 210–213, 215–229, 235–239, 654.1, 654.10, 654.11, 654.12, 654.13, and 654.14), coronary artery disease (ICD-9-CM codes 410–413, 414.01–414.05, 414.8, and 414.9), hyperlipidemia (ICD-9-CM code 272), hypertension (ICD-9-CM codes 401–405), congestive heart failure (ICD-9-CM codes 428, 398.91, and 402.x1),atrial fibrillation (ICD-9-CM code 427.31), stroke (ICD-9-CM codes 430–438), chronic hepatitis (ICD-9-CM codes 571, 572.2, 572.3, 572.8, 573.1, 573.2, 573.3, 573.8, and 573.9), hypoglycemia (ICD-9-CM codes 251), acute respiratory infections (ICD-9-CM codes 460–466), other diseases of the upper respiratory tract (ICD-9-CM codes 470–478), and pneumonia (ICD-9-CM codes 480–486) are identified as comorbidities before the index date.

### Statistical analysis

For every individual patient, GV was measured as coefficient of variation (CV) of FPG and HbA1c calculated from first-year datasets of electronic laboratory records. The patients were grouped in terms of tertiles of FPG-CV (first tertile: ≤ 17.3%, second tertile: 17.3% to 34.6%, and third tertile: > 34.6%) and HbA1c-CV (first tertile: ≤ 8.4%, second tertile: 8.4% to 17.1%, and third tertile: > 17.1%). Baseline characteristics among HbA1c-CV tertile group were compared using standardized mean differences, which were calculated as the mean or proportion difference of a variable divided by the pooled standard deviation of the variable [26]. This measure is not influenced by sample size and is useful for comparison between cohorts with large size of sample in observational studies. A value of 0.1 or less indicates a negligible difference in means or proportions between groups. The extended Cox proportional hazards model based on the Lunn–McNeil approach was performed as the modified model considering competing risks with multivariable adjustment. The extended Cox proportional hazards models with the competing risk of all-cause death were used to investigate the association between GV (FPG-CV and HbA1c-CV) and risk of incident COPD. The hazard ratios (HRs) and their 95% confidence intervals (CI) were calculated by using three hierarchical multivariate models. The first multivariate model was adjusted for age (continuous) and gender (male or female). The second one was additionally adjusted for variables as follows: duration of diabetes (continuous, in years), smoking (yes or no), alcohol consumption (yes or no), hypertension (yes or no), antihypertensive treatment (yes or no), obesity (yes or no), baseline FPG and HbA1c levels, and type of hypoglycemic drug [no medication, metformin monotherapy, sulfonylurea monotherapy, and oral antidiabetic drug (OAD) monotherapy other than metformin or sulfonylurea (OAD-other), combination of metformin plus sulfonylurea, combination of metformin plus OAD-other, combination of sulfonylurea plus OAD-other, combination of 2 OAD-other, combination of 3 OAD, combination of more than 3 OAD, insulin monotherapy, insulin plus one OAD, insulin plus more than one OAD, and short-time OAD; OAD-other consisted of α-glucosidase inhibitors, thiazolidinedione, and meglitinide]. The third model was additionally adjusted for cancer (yes or no), coronary artery diseases (yes or no), congestive heart failure (yes or no), hyperlipidemia (yes or no), atrial fibrillation (yes or no), stroke (yes or no), chronic hepatitis (yes or no), diabetic retinopathy (yes or no), hypoglycemia (yes or no), and acute respiratory infections (yes or no), other diseases of the upper respiratory tract (yes or no), and pneumonia (yes or no). Additional analysis were conducted using propensity-score matching being generated by multinomial logistic regression using HbA1c-CV tertile group as dependent variable and covariates in the third model as independent variables. The propensity-score match approach was performed to balance covariate distributions among 3 HbA1c-CV tertile groups. The stratified Cox model was applied to calculate HR for propensity-score matched cohort. The *P* value was calculated for evaluating the trend across the tertiles of FPG-CV and HbA1c-CV, and the proportionality assumption was examined by including an interaction term of tertiles of FPG-CV or HbA1c-CV with person-time into the Cox models. We found no statistically significant violation in this assumption. Sensitivity analysis was further performed by excluding patients with severe chronic disorders or complications to evaluate potential biases caused by existence of comorbidity. All analyses were performed using SAS version 9.4 (SAS, Cary, NC). All *P*-values were two-tailed, and *P* < 0.05 was considered as statistically significant.

## Results

During an average of 7.58 years of follow-up, 2,346 incidence cases of COPD were identified in the present diabetic cohort; these cases correspond to a crude incidence rate of 11.36 per 1,000 person-years (12.90 for men and 10.03 for women). Table 1 shows the baseline sociodemographic and clinical characteristics of the patients grouped according to HbA1c-CV tertile groups. Before propensity-score matching, patients in the highest HbAc1-CV tertile group had younger mean age, shorter mean duration of diabetes, lower prevalence of one oral hypoglycemic drug, hypertension drug treatment, hyperlipidemia, hypertension, higher prevalence of insulin plus oral hypoglycemic drug, and higher mean baseline HbA1c and FPG as well as FPG-CV (Table 1). The distributions of these characteristics were in general similar across HbA1c-CV tertile groups after propensity-score matching were conducted (S2 Table).

**Table 1:** The comparisons of baseline sociodemographic factors, life style behaviors, diabetes-related variables, drug-related variables and co-morbidity according to HbA1c-CV tertile groups in patients with type 2 diabetes enrolled in the National Diabetes Care Management Program, Taiwan (N = 27,257).

Variables	HbA1c-CV (%)					
	Full cohort (N = 27,257)			Propensity-score matched (N = 16,530)		
	≤8.4% (n = 9,020)	8.4%-17.1% (n = 9,005)	>17.1% (n = 9,232)	≤8.4% (n = 5,510)	8.4%-17.1% (n = 5,510)	>17.1% (n = 5,510)
*Sociodemographic factors*						
Gender						
Female	4934 (54.70)	4884 (54.24)	4691 (50.81)	2958 (53.68)	3012 (54.66)	2945 (53.45)
Male	4086 (45.30)	4121 (45.76)	4541 (49.19)	2552 (46.32)	2498 (45.34)	2565 (46.55)
Age (years)	60.93 (10.90)	60.77 (10.99)	59.54 (11.39)	60.32 (10.83)	60.81 (10.91)	59.98 (11.29)
*Lifestyle behaviors*						
Smoking	1253 (13.89)	1276 (14.17)	1626 (17.61)	843 (15.30)	775 (14.07)	879 (15.95)
Alcohol drinking	708 (7.85)	780 (8.66)	890 (9.64)	477 (8.66)	462 (8.38)	496 (9.00)
*Diabetes-related variables*						
Duration of diabetes (years)	7.11 (8.16)	7.00 (7.10)	5.79 (6.90)	6.74 (6.45)	7.11 (6.92)	6.22 (7.00)
Type of hypoglycemic drug use						
No medication	156 (1.73)	85 (0.94)	90 (0.97)	38 (0.69)	28 (0.51)	56 (1.02)
One oral hypoglycemic drug	1958 (21.71)	1422 (15.79)	1373 (14.87)	770 (13.97)	719 (13.05)	889 (16.13)
Two oral hypoglycemic drugs	3918 (43.44)	3794 (42.13)	3896 (42.20)	2454 (44.54)	2350 (42.65)	2357 (42.78)
Three oral hypoglycemic drugs	1500 (16.63)	1728 (19.19)	1800 (19.50)	1098 (19.93)	1132 (20.54)	1044 (18.95)
>3 oral hypoglycemic drugs	399 (4.42)	472 (5.24)	581 (6.29)	316 (5.74)	302 (5.48)	331 (6.01)
Insulin	240 (2.66)	275 (3.05)	199 (2.16)	147 (2.67)	187 (3.39)	153 (2.78)
Insulin+ oral hypoglycemic drug	849 (9.41)	1229 (13.65)	1293 (14.01)	687 (12.47)	792 (14.37)	680 (12.34)
*Drug-related variables*						
Hypertension drug treatment	3713 (41.16)	3457 (38.39)	3083 (33.39)	2016 (36.59)	2083 (37.80)	1966 (35.68)
Glucocorticoids	142 (1.57)	136 (1.51)	154 (1.67)	85 (1.54)	81 (1.47)	88 (1.60)
*Comorbidity*						
Obesity	3354 (37.18)	3328 (36.96)	3196 (34.62)	1988 (36.08)	2049 (37.19)	1986 (36.04)
CAD	737 (8.17)	751 (8.34)	625 (6.77)	411 (7.46)	461 (8.37)	384 (6.97)
CHF	151 (1.67)	189 (2.10)	182 (1.97)	109 (1.98)	131 (2.38)	96 (1.74)
Cancer	167 (1.85)	168 (1.87)	181 (1.96)	107 (1.94)	92 (1.67)	107 (1.94)
Hyperlipidemia	2669 (29.59)	2444 (27.14)	2167 (23.47)	1437 (26.08)	1477 (26.81)	1405 (25.50)
Hypertension	4187 (46.42)	4104 (45.57)	3651 (39.55)	2388 (43.34)	2519 (45.72)	2353 (42.70)
Atrial fibrillation	35 (0.39)	37 (0.41)	35 (0.38)	17 (0.31)	24 (0.44)	18 (0.33)
Chronic hepatitis	857 (9.50)	935 (10.38)	886 (9.60)	548 (9.95)	601 (10.91)	539 (9.78)
Diabetic retinopathy	2015 (22.34)	2104 (23.36)	2089 (22.63)	1314 (23.85)	1308 (23.74)	1256 (22.79)
Hypoglycemia	27 (0.30)	26 (0.29)	29 (0.31)	19 (0.34)	15 (0.27)	17 (0.31)
Pneumonia	62 (0.69)	70 (0.78)	78 (0.84)	47 (0.85)	45 (0.82)	39 (0.71)
Other diseases of the upper respiratory tract	92 (1.02)	75 (0.83)	78 (0.84)	48 (0.87)	50 (0.91)	50 (0.91)
Acute respiratory infections	618 (6.85)	592 (6.57)	555 (6.01)	382 (6.93)	369 (6.7)	351 (6.37)
*Blood biochemical measurement*						
HbA1c (%)	7.59 (1.57)	7.96 (1.52)	8.39 (1.50)	8.09 (1.66)	8.06 (1.51)	8.12 (1.40)
Fasting plasma glucose (mg/dL)	158.63 (46.26)	167.27 (46.18)	177.09 (50.66)	169.74 (50.63)	169.73 (46.18)	170.93 (48.74)
FPG-CV (%)	24.42 (21.24)	29.31 (22.08)	41.64 (30.35)	31.79 (23.37)	31.21 (21.47)	33.57 (26.62)

Data were presented as counts (percentage) for categorical variables and mean (SD) for continuous variables.

SU: sulfonylurea; Met: metformin; Meg: meglitinide; Big: biguanide; TZD: thiazolidinedione; CAD: coronary artery disease; CHF: congestive heart failure; COPD: chronic obstructive pulmonary disease; FPG: fasting plasma glucose; FPG-CV: coefficient of variation of fasting plasma glucose; HbA1c-CV: coefficient of variation of HbA1c; SD: standard deviation.

**Fig 2:**
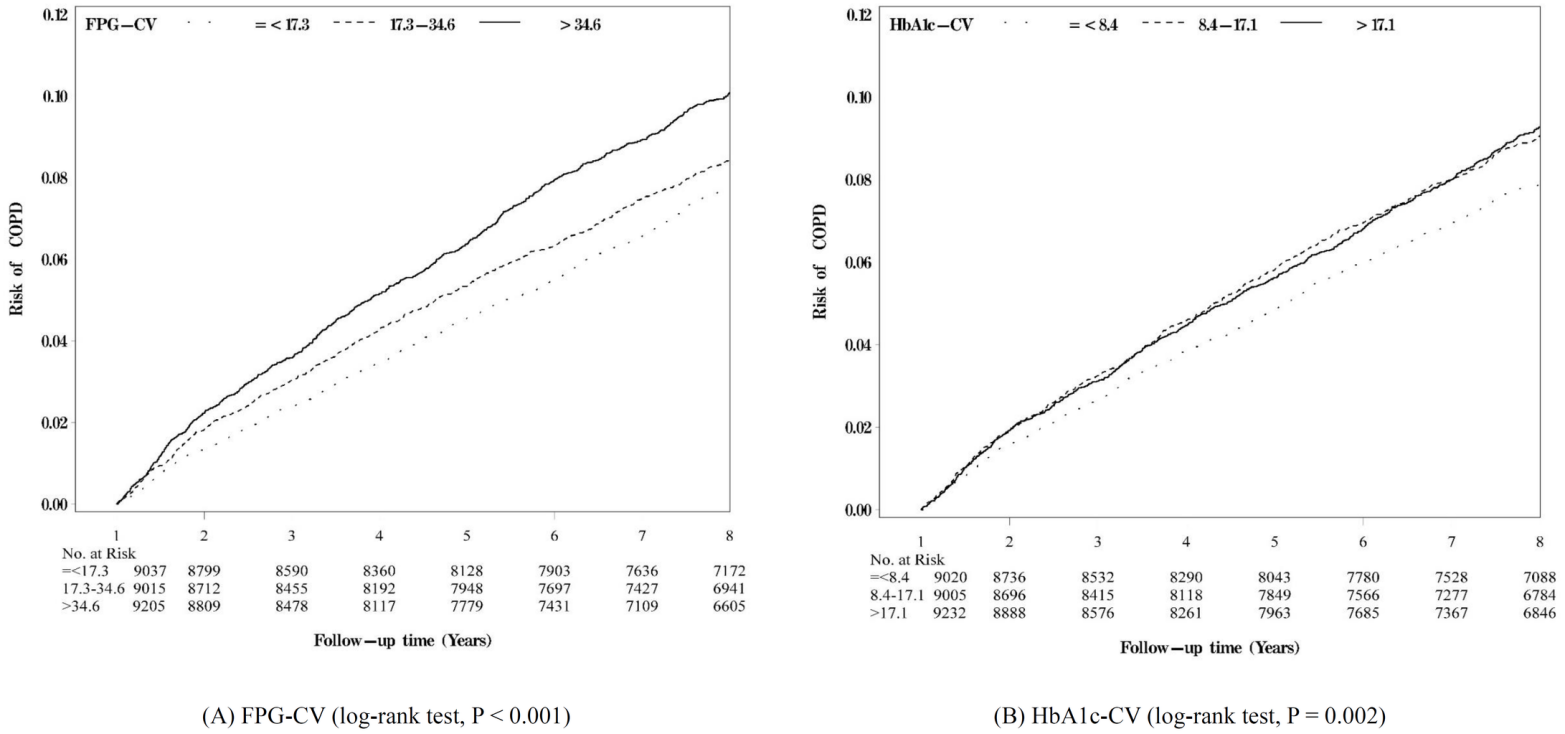
Kaplan-Meier cumulative incidence curves of COPD according to tertiles of (A) FPG-CV and (B) HbA1c-CV, respectively. FPG-CV: coefficient of variation of fasting plasma glucose; HbA1c-CV: coefficient of variation of HbA1c.

Fig 2 shows the Kaplan–Meier cumulative incidence curves of COPD within the subgroups defined by tertiles of FPG-CV (Fig 2A) and HbA1c-CV (Fig 2B), respectively. Patients within the subgroups of second and third tertiles of FPG-CV exhibited a higher risk of developing COPD than those of the first tertile of FPG-CV (log-rank test, *P* < 0.001) (Fig 2A). Similarly, patients within the subgroups of second and third tertiles of HbA1c-CV showed a higher risk of developing COPD than those of the first tertile of HbA1c-CV (log-rank test, *P* = 0.002) (Fig 2B). The differences in cumulative incidence curves of COPD among the subgroups of three tertiles seemed more apparent for FPG-CV than those for HbA1c-CV.

Table 2 presents the HRs of COPD among patients grouped by HbA1c levels and tertiles of FPG-CV or/and HbA1c-CV. The incidence rates in the first, second and third tertiles were 9.87, 11.06, and 13.19 per 1000 person–years, respectively, for FPG-CV and 10.02, 11.81, and 12.07 per 1000 person–years, respectively, for HbA1c-CV. In the full cohort, compared with patients of the first tertile of FPG-CV, the age–gender adjusted HRs of COPD were 1.43 (95% CI: 1.30 to 1.58) and 1.16 (95% CI: 1.05 to 1.28) in patients of the third and second tertiles, respectively. Likewise, patients of the third and second tertiles of HbA1c-CV showed increased age–gender adjusted HRs of COPD (third tertile: 1.29, 95% CI: 1.17 to 1.43; second tertile: 1.20, 95% CI: 1.09 to 1.33) compared with those in the first tertile. The trends of HRs of COPD across the three tertiles were linear for both FPG-CV and HbA1c-CV (*P* for trend < 0.001). Subsequent analysis considered age, gender, diabetes duration, treatment type, smoking, alcohol consumption, obesity, baseline FPG and HbA1c levels, comorbidities and complications, and mutual adjustment for FPG-CV and HbA1c-CV. The results showed that the effects of FPG-CV and HbA1c-CV were slightly attenuated but remained statistically significant for the third versus the first tertile of FPG-CV (1.26, 95% CI: 1.13 to 1.40) as well as for the third or second versus the first tertile of HbA1c-CV (1.13, 95% CI: 1.02 to 1.26; 1.13, 95% CI: 1.02 to 1.25). Furthermore, the linear trend of HRs of COPD across the three tertiles remained significant for FPG-CV (*P* for trend < 0.001) and HbA1c-CV (*P* for trend = 0.02) after multivariate adjustment. In addition, the association between FPG-CV tertiles and HbA1c-CV tertiles and risk of COPD after propensity matching remains similar to the results found in the full cohort. Compared to those with HbA1c of 6~7, the multivariate-adjusted HRs considering HbA1c-CV and FPG-CV simultaneously were 1.22 (95% CI: 1.04 to 1.42), and 1.22 (95% CI: 0.961.04 to 1.41) in the full cohort, for those with HbA1c of <6, and > 10, respectively. These significant HRs of HbA1c level became non-significant after propensity score matching.

**Table 2:** The hazard ratios (HRs) of chronic obstructive pulmonary disease according to tertiles of FPG-CV and HbA1c-CV in diabetic patients enrolled in the National Diabetes Care Management Program, Taiwan.

	COPD HR (95% CI)					
	Unmatched population (N = 27,257)			Matched population (N = 16,530)		
Variables	Age & gender-adjusted	Multivariate- Adjusted^1^	Multivariate- Adjusted^2^	Age & gender-adjusted	Multivariate- Adjusted^1^	Multivariate- Adjusted^2^
FPG-CV (%)						
≤17.3	1.00	1.00	1.00	1.00	1.00	1.00
17.3–34.6	1.13 (1.02–1.25)*	1.11 (1.00–1.23)	1.11 (1.00–1.23)	1.07 (0.94–1.23)	1.05 (0.92, 1.20)	1.05 (0.92, 1.20)
>34.6	1.36 (1.22–1.50)***	1.28 (1.16–1.42)***	1.28 (1.16–1.42)***	1.31 (1.14–1.50)***	1.24 (1.08, 1.43)**	1.25 (1.09, 1.44)**
P for trend	<0.001	<0.001	<0.001	<0.001	0.001	<0.001
HbA1c (%)						
<6	1.23 (1.05–1.43)*	1.24 (1.06–1.45)**	1.22 (1.05–1.43)*	1.22 (0.97–1.52)	1.20 (0.96–1.50)	1.18 (0.94–1.48)
6~7	1.00	1.00	1.00	1.00	1.00	1.00
7~8	1.10 (0.97–1.25)	1.05 (0.93–1.2)	1.05 (0.93–1.20)	1.09 (0.93–1.28)	1.03 (0.87–1.21)	1.03 (0.87–1.21)
8~9	1.16 (1.02–1.33)*	1.08 (0.94–1.24)	1.08 (0.94–1.24)	1.19 (1.01–1.41)*	1.08 (0.91–1.29)	1.09 (0.91–1.29)
9~10	1.23 (1.06–1.42)**	1.08 (0.93–1.27)	1.09 (0.93–1.28)	1.36 (1.13–1.63)**	1.17 (0.96–1.43)	1.17 (0.96–1.43)
≥10	1.43 (1.26–1.63)***	1.23 (1.05–1.43)**	1.24 (1.07–1.44)**	1.46 (1.22–1.73)***	1.19 (0.97–1.45)	1.20 (0.98–1.46)
HbA1c-CV (%)						
≤8.4	1.00	1.00	1.00	1.00	1.00	1.00
8.4–17.1	1.17 (1.06–1.29)**	1.15 (1.04–1.27)**	1.15 (1.03–1.27)**	1.16 (1.02–1.33)*	1.15 (1.01–1.31)*	1.15 (1.01–1.31)*
>17.1	1.19 (1.07–1.32)**	1.17 (1.06–1.31)**	1.17 (1.05–1.30)**	1.13 (0.99–1.29)	1.15 (1.01–1.30)*	1.15 (1.01–1.31)*
P for trend	<0.001	<0.001	<0.001	0.02	0.02	0.02
HbA1c (%)						
<6	1.21 (1.03–1.41)*	1.23 (1.05–1.44)**	1.21 (1.04–1.42)*	1.20 (0.96–1.50)	1.19 (0.95–1.48)	1.17 (0.93–1.46)
6~7	1.00	1.00	1.00	1.00	1.00	1.00
7~8	1.10 (0.98–1.25)	1.05 (0.93–1.20	1.05 (0.93–1.19)	1.08 (0.92–1.27)	1.02 (0.87–1.20)	1.02 (0.86–1.20)
8~9	1.17 (1.03–1.34)*	1.08 (0.94–1.24)	1.07 (0.93–1.23)	1.17 (0.99–1.39)	1.06 (0.89–1.27)	1.06 (0.89–1.27)
9~10	1.24 (1.07–1.44)**	1.08 (0.93–1.27)	1.09 (0.93–1.27)	1.33 (1.10–1.6)**	1.14 (0.94–1.39)	1.14 (0.93–1.39)
≥10	1.47 (1.29–1.68)***	1.23 (1.05–1.43)**	1.24 (1.07–1.45)**	1.44 (1.21–1.72)***	1.16 (0.95–1.42)	1.17 (0.96–1.44)
FPG-CV (%)						
≤17.3	1.00	1.00	1.00	1.00	1.00	1.00
17.3–34.6	1.12 (1.00–1.24)*	1.09 (0.98–1.21)	1.09 (0.98–1.21)	1.07 (0.93–1.22)	1.05 (0.92–1.20)	1.05 (0.92–1.2)
>34.6	1.33 (1.20–1.47)***	1.26 (1.13–1.40)***	1.26 (1.13–1.40)***	1.31 (1.14–1.50)***	1.25 (1.08–1.43)**	1.25 (1.09–1.45)**
P for trend	<0.001	<0.001	<0.001	<0.001	0.001	<0.001
HbA1c-CV (%)						
≤8.4	1.00	1.00	1.00	1.00	1.00	1.00
8.4–17.1	1.14 (1.03–1.26)*	1.13 (1.02–1.25)*	1.12 (1.02–1.25)*	1.17 (1.02–1.33)*	1.16 (1.01–1.32)*	1.16 (1.02–1.32)*
>17.1	1.11 (1.00–1.24)*	1.11 (1.00–1.24)	1.11 (1.00–1.24)	1.14 (1.00–1.30)	1.15 (1.01–1.31)*	1.15 (1.01–1.31)*
P for trend	0.01	0.02	0.02	0.01	0.02	0.02
HbA1c (%)						
<6	1.22 (1.04–1.42)*	1.23 (1.05–1.44)**	1.22 (1.04–1.42)*	1.20 (0.96–1.49)	1.18 (0.95–1.47)	1.16 (0.93–1.45)
6~7	1.00	1.00	1.00	1.00	1.00	1.00
7~8	1.09 (0.96–1.24)	1.05 (0.92–1.19)	1.05 (0.92–1.19)	1.08 (0.92–1.27)	1.02 (0.87–1.21)	1.02 (0.87–1.21)
8~9	1.14 (1.00–1.30)	1.06 (0.93–1.22)	1.06 (0.92–1.22)	1.17 (0.99–1.39)	1.07 (0.90–1.27)	1.07 (0.90–1.28)
9~10	1.19 (1.03–1.39)*	1.07 (0.91–1.25)	1.07 (0.91–1.26)	1.33 (1.10–1.60)**	1.15 (0.95–1.41)	1.15 (0.94–1.40)
≥10	1.39 (1.22–1.60)***	1.20 (1.03–1.40)*	1.22 (1.04–1.41)*	1.42 (1.19–1.70)***	1.16 (0.95–1.42)	1.17 (0.96–1.43)

*:p<0.05;

**:P<0.01;

***:p<0.001.

Multivariate-adjusted^1^ for age, gender, smoking, alcohol consumption, duration of diabetes, type of hypoglycemic drugs, hypertension drug treatment, use of glucocorticoids, obesity, baseline fasting glucose and HbA1c.

Multivariate-adjusted^2^ for coronary artery disease, congestive heart failure, cancer, hyperlipidemia, hypertension, atrial fibrillation, chronic hepatitis, diabetic retinopathy, hypoglycemia, pneumonia, other diseases of the upper respiratory tract and acute respiratory infections in addition to the variables in the first multivariate model.

IR: incidence density rate = number of incident cases / person-years*1000.

FPG-CV: coefficient of variation of fasting plasma glucose; HbA1c-CV: coefficient of variation of HbA1c; HR: hazard ratio.

### Sensitivity analysis

Table 3 demonstrates the results of sensitivity analysis conducted to rule out potential bias caused by existence of comorbidities by excluding patients with hyperglycemic hyperosmolar nonketotic coma, diabetic ketoacidosis, coronary artery disease, chronic hepatitis, and hypoglycemia (n = 22,133 for sensitivity analysis). The hazard ratio of COPD for the third versus the first tertile of FPG-CV was 1.22 (1.08–1.38), whereas that for the third versus the first tertile of HbA1c-CV was 1.15 (1.02–1.29). In order assess whether our results are sensitive to cutoff points of glucose variation in FPG and HbA1c, quintiles of FPG-CV and HbA1c-CV were used instead of tertiles. With FPG-CV subgrouped based on quintile, multivariate-adjusted HRs for FPG-CV levels from 4^th^ to 5^th^ quintile were 1.18 (1.03, 1.35), and 1.31 (1.15, 1.49), respectively; and for HbA1c-CV were 1.14 (1.00, 1.31), 1.15 (1.01, 1.31), 1.32 (1.16, 1.51), and 1.20 (1.05, 1.38), respectively.

**Table 3:** Sensitivity analysis for evaluating bias because of comorbidity by excluding patients with hyperglycemic hyperosmolar nonketotic coma, diabetic ketoacidosis, myocardial infarction, atrial fibrillation, and hypoglycemia.

		COPD HR (95% CI)					
		FPG-CV (%)			HbA1c-CV (%)		
	N	≤ 17.3	17.3–34.6	> 34.6	≤ 8.4	8.4–17.1	> 17.1
Model I	26,830	1.00	1.10 (0.99–1.22)	1.26 (1.13–1.40)***	1.00	1.14 (1.02–1.26)*	1.14 (1.02–1.27)*
Model II	26,991	1.00	1.08 (0.97–1.20)	1.25 (1.13–1.40)***	1.00	1.13 (1.02–1.25)*	1.14 (1.03–1.27)*
Model III	25,144	1.00	1.08 (0.97–1.21)	1.24 (1.11–1.39)***	1.00	1.11 (1.00–1.24)	1.14 (1.02–1.28)*
Model IV	24,579	1.00	1.10 (0.99–1.23)	1.26 (1.12–1.40)***	1.00	1.10 (0.99–1.22)	1.12 (1.00–1.25)*
Model V	27,175	1.00	1.10 (0.99–1.22)	1.26 (1.14–1.41)***	1.00	1.12 (1.01–1.25)*	1.13 (1.02–1.26)*
Model VI	22,133	1.00	1.09 (0.97–1.22)	1.23 (1.09–1.38)**	1.00	1.08 (0.96–1.22)	1.15 (1.02–1.29)*

*:p<0.05;

**:P<0.01;

***:p<0.001.

Multivariate adjusted for age, gender, smoking, alcohol consumption, duration of diabetes,type of hypoglycemic drug, hypertension drug treatment, use of glucocorticoids, obesity, coronary artery disease, congestive heart failure, cancer, hyperlipidemia, hypertension, atrial fibrillation, diabetic retinopathy, hypoglycemia, pneumonia, other diseases of the upper respiratory tract and acute respiratory infections, baseline fasting glucose, and HbA1c.

Model I excluding patients with hyperglycemic hyperosmolar nonketotic coma (HHNK) (N = 427).

Model II excluding patients with diabetic ketoacidosis (DKA) (N = 266).

Model III excluding patients with coronary artery disease (N = 2113).

Model IV excluding patients with chronic hepatitis (N = 2678).

Model V excluding patients with hypoglycemia (N = 82).

Model VI excluding patients with HHNK, DKA, coronary artery disease, chronic hepatitis, and hypoglycemia. (N = 5124).

## Discussion

This study showed that baseline GV measured by visit-to-visit variability in FPG and HbA1c is independently associated with incident COPD among patients with type 2 diabetes. The risk of COPD is higher among those with FPG-CV in the top tertile (> 34.6%) or HbA1c-CV in the second or third tertile (> 8.4%) compared with those with glycemic variability in the lowest tertile. The findings regarding associations between FPG-CV or HbA1c-CV and COPD incidence remain consistent after excluding patients with severe comorbidities or complications; hence, the association between GV and COPD is robust among type 2 diabetic population. These results provide insights into clinical management of patients with type 2 diabetes to monitor oscillation of blood glucose and prevent lung dysfunction.

Diabetes and COPD are chronic diseases with great disease burden, which rank seventh and eighth leading causes of global YLDs, respectively [1]. The relationship between diabetes and COPD are complex and controversial [27]. A previous population-based longitudinal cohort study revealed that patients diagnosed with diabetes were at elevated risk of several pulmonary diseases, including COPD [28]. Chronic systemic inflammation could be the plausible mechanism to explain the observed association between type 2 diabetes and risk of COPD. Type 2 diabetes and COPD development are related to chronic systemic inflammation caused by the presence of metabolic disorders or cardiovascular diseases [29, 30]. Experimental studies suggested that hyperglycemia could induce inflammatory responses [31], which could eventually result in structural changes in lung tissues and impaired pulmonary function. In addition, *in vivo* studies confirmed that hyperglycemia could lead to structural modifications via oxidative stress in lung tissues and restricted gas exchange [32]. Observational studies revealed an evident correlation between reduced lung function and unbalanced blood glucose [17–21]. Similarly, pathological studies in humans showed that the epithelia and capillary basal membranes of the alveoli were profoundly thicker in diabetics compared with those in age-matched controls [33]. These findings implied that hyperglycemia may induce inflammatory responses, which could eventually result in harmful alterations in lung tissues and insufficient pulmonary function.

In this study, we examined the association between GV and risk of COPD among patients with type 2 diabetes. We found that high GV is associated with elevated risk of COPD. GV is a measure that accounts for the amplitude, frequency, and duration of glycemic oscillations around the average blood glucose level [6]. The episodes of either hyper- or hypoglycemia could also contribute to the calculation of GV. In our previous work, baseline extreme HbA1c values were demonstrated to be associated with increased risk of COPD among patients with type 2 diabetes [23]; hence, either hyper- or hypoglycemia is a factor that affects progression of respiratory disorders among diabetic patients. Nevertheless, the association between baseline HbA1c values and risk of COPD was not significant in the propensity-score matched sample after considering HbA1c-CV and FPG-CV in the multivariate analysis. The lack of significance for baseline HbA1c after propensity score matching may be due to smaller sample size. Given the sample size, distribution of HbA1c, and strength of association between HbA1c and COPD in our propensity-score matched sample, the power was 0.56. On the contrary, the power of our prior study reporting the association between HbA1c and COPD was 0.94.

Previous studies reported the associations between acute hyperglycemia with a spectrum of poor outcomes, including myocardial infraction [34], stroke [35], and pneumonia [36]. Hypoglycemia also adversely influenced chronic kidney diseases [37], cardiovascular events [38], and dementia [39]. These studies revealed the potential harmful effects of fluctuations of blood glucose on diabetic patients. Furthermore, some experimental studies discovered the process through which glucose oscillations exacerbate the adverse effects of constant hyperglycemia on endothelial cells and inflammatory cytokines via oxidative stress [31, 40–42]. Although the linkage between type 2 diabetes and COPD is supported by an epidemiologic study [28], the present study provides further evidence that the fluctuation of blood glucose in patients with type 2 diabetes may play a pivotal role on disease progression.

In this study, FPG-CV and HbA1c-CV are predictors for COPD incidence, and the former showed more potent effect on COPD relative to HbA1c-CV. Based on the magnitude of the strength of association, FPG-CV seems to be a more effective indicator than HbA1c-CV. HbA1c reflects an approximately 3-month average of glycemic status [8]; thus, the values for variations in routine HbA1c records are smaller relative to those in FPG. Assuming that COPD relationship to variations in GV is fixed, then the variation in FPG can be used to easily capture the variations in COPD incidence because of its larger variation. FPG level can be used to determine acute oscillations of blood glucose caused by irregular diet or lifestyle episode; such oscillations cannot be easily detected by HbA1c test. FPG-CV is a more sensitive indicator than HbA1c for capturing additional variations in glucose caused by overindulgence in food during social events. Thus, FPG-CV is a promising measure of GV given that HbA1c is considered a mean.

To the extent of our knowledge, this study is the first population-based, long-term cohort study that investigated the relationship between GV and COPD among patients with type 2 diabetes. A sample size of around 27,000 is relatively large and more powerful compared with relevant observational and epidemiologic studies. In contrast to other diabetes studies using population-based healthcare databases [28], the present study showed that the database used, which includes data on FPG and HbA1c, can be used for GV calculation. The application of competing risk analysis considering all-cause death avoids biased estimates with regard to the association between GV and COPD. Moreover, multivariate-adjusted models and additional sensitivity analysis consolidate the robustness of the findings. However, several limitations should be considered. First, residual or unknown confounders may influence the estimated association between GV and COPD because of the observational study design. Second, GV in the current study is merely measured as CV of at least two blood glucose records within one-year time frame. Thus, we could not evaluate annual variation in FPC and HbA1c, which reflects intra-individual variation in follow-up years, on the risk of COPD. Third, the database does not contain information on lung function, thus we don’t know how well study subjects’ lungs are functioning. We also can’t evaluate the status of restrictive lung diseases. In addition to COPD, lung function and restrictive lung diseases are also important aspects of health in lung. Unlike COPD, restrictive disease are associated with a decreased total lung capacity. Future studies assessing lung function to explore the relationships between glucose variation and lung function or restricted lung diseases are warranted. Finally, our study only provides the results of FPG-CV and HbA1c -CV relationship to COPD risk. The assessment of glucose variability is complex and has been quantified by many alternative ways, including within-day blood glucose variation, pre- and post-prandial glucose, and hypoglycemia and hyperglycemia episodes. This study did not measure either within-day blood glucose variation or post-prandial glucose and thus could not assess the effects of these indicators on COPD risk. No “gold standard” currently exists to rate glucose variability. Future studies must compare the relative predictive capacity of glucose variation indicators for COPD incidence to estimate COPD risk in clinical practice. Our data showed both FPG-CV and HbA1c -CV predict COPD in patients with type 2 DM. Furthermore, visit-to-visit variability in FPG and HbA1c levels as a measure of GV is a feasible approach and thus can be used in clinical practice.

## Conclusions

In conclusion, in this large cohort of Chinese patients with type 2 diabetes, FPG-CV and HbA1c -CV were independent predictors of COPD after adjusting for mean FPG, HbA_1c_, and other conventional risk factors. The findings provide implications for diabetes management in terms of monitoring GV, especially by using FPG-CV and HbA1c-CV in clinical practice in addition to conventional glycemic measurements. For patients with wide oscillations of blood glucose, glucose management should be the focus to prevent the risk of lung dysfunction and probable COPD incidence.

## Supporting information

S1 Table Comparisons of baseline sociodemographic factors, lifestyles, diabetes-related variables, drug-related variables, comorbidity and blood biochemical measurement between patients included and excluded (n = 48,354). (PDF)

S2 Table Standardized mean differences of baseline sociodemographic factors, life style behaviors, diabetes-related variables, drug-related variables, comorbidity and blood biochemical measurement among tertile groups of HbA1c-CV in patients with type 2 diabetes enrolled in the National Diabetes Care Management Program, Taiwan. (PDF)

## Abbreviations

ADA: American Diabetes Association
CI: confidence interval
COPD: chronic obstructive pulmonary disease
CV: coefficient of variation
CVD: cardiovascular disease
DM: diabetes mellitus
FEV1: forced expiratory volume in 1 second
HbA1c: glycated hemoglobin
HR: hazard ratio
ICD-9-CM: International Classification of Disease, 9th Revision, Clinical Modification
NDCMP: National Diabetes Case Management Program
NHI: National Health Insurance
NHIRD: National Health Insurance Research Database
OAD: oral antidiabetic drug
PIN: personal identification number
