# Supplementary material for: Visit-to-visit glycemic variability is a strong predictor of chronic obstructive pulmonary disease in patients with type 2 diabetes mellitus: Competing risk analysis using a national cohort from the Taiwan diabetes study
Source: PLoS One. 2017 May 10;12(5):e0177184. doi: 10.1371/journal.pone.0177184 (PMC5425194; doi:10.1371/journal.pone.0177184)
Supplement: S1 Table — (PDF) [file pone.0177184.s001.pdf]

S1 Table. Comparisons of baseline sociodemographic factors, lifestyles, diabetes-related variables, drug-related variables, comorbidity and blood biochemical measurement between patients included and excluded (n=48,354)

Variables	No (%) Excluded patients (N=21,097)	Included patients (N=27,257)	Standardized Mean Difference
Sociodemographic factors			
Gender			
Female	10899 (51.78)	14509 (53.23)	-0.03
Male	10151 (48.22)	12748 (46.77)	0.03
Age (years)	58.90 (15.91)	60.41 (11.11)	-011
Lifestyle behaviors			
Smoking	3530 (16.77)	4155 (15.24)	0.04
Alcohol drinking	1922 (9.13)	2378 (8.72)	0.01
Diabetes-related variables			
Duration of diabetes (years)	6.24 (15.44)	6.63 (7.43)	-0.03
Type of hypoglycemic drug use			
No medication	1226 (5.81)	331 (1.21)	0.26
One oral hypoglycemic drug	4220 (20.00)	4753 (17.44)	0.07
Two oral hypoglycemic drugs	8622 (40.87)	11608 (42.59)	-0.03
Three oral hypoglycemic drugs	3364 (15.95)	5028 (18.45)	-0.07
>3 oral hypoglycemic drugs	930 (4.41)	1452 (5.33)	-0.04
Insulin	550 (2.61)	714 (2.62)	0.00
Insulin+ oral hypoglycemic drug	2185 (10.36)	3371 (12.37)	-0.06
Drug-related variables			
Hypertension drug treatment	6979 (33.08)	10253 (37.62)	-0.09
Glucocorticoids	277 (1.31)	432 (1.58)	-0.02
Comorbidity			
Obesity	6962 (34.92)	9878 (36.24)	-0.03
CAD	1570 (7.44)	2113 (7.75)	-0.01
CHF	356 (1.69)	522 (1.92)	-0.02
Cancer	440 (2.09)	516 (1.89)	0.01
Hyperlipidemia	4817 (22.83)	7280 (26.71)	-0.09
Hypertension	8169 (38.72)	11942 (43.81)	-0.10
Atrial fibrillation	74 (0.35)	107 (0.39)	-0.01
Chronic hepatitis	1945 (9.22)	2678 (9.82)	-0.02
Diabetic retinopathy	4618 (21.89)	6208 (22.78)	-0.02
Hypoglycemia	52 (0.25)	82 (0.30)	-0.01
Pneumonia	176 (0.83)	210 (0.77)	0.01
Other diseases of the upper respiratory tract	139 (0.66)	245 (0.90)	-0.03
Acute respiratory infections	1077 (5.10)	1765 (6.48)	-0.06
Blood biochemical measurement			
Fasting plasma glucose	166.90 (54.77)	167.74 (48.36)	-0.02
HbA1c	8.01 (1.61)	7.98 (1.56)	0.02
FPG-CV	37.03 (30.87)	31.87 (25.98)	0.20
HbA1c-CV	19.80 (17.50)	16.64 (15.20)	0.20

CAD: coronary artery disease; CHF: congestive heart failure; FPG-CV: coefficient of variation of fasting plasma glucose; HbA1c-CV: coefficient of variation of HbA1c.

a: A value of 0.1 or less indicates a negligible difference in means or proportions.
