# Supplementary material for: Visit-to-visit glycemic variability is a strong predictor of chronic obstructive pulmonary disease in patients with type 2 diabetes mellitus: Competing risk analysis using a national cohort from the Taiwan diabetes study
Source: PLoS One. 2017 May 10;12(5):e0177184. doi: 10.1371/journal.pone.0177184 (PMC5425194; doi:10.1371/journal.pone.0177184)
Supplement: S2 Table — (PDF) [file pone.0177184.s002.pdf]

S2 Table. Standardized mean differences of baseline sociodemographic factors, life style behaviors, diabetes-related variables, drug-related variables, comorbidity and blood biochemical measurement among tertile groups of HbA1c-CV in patients with type 2 diabetes enrolled in the National Diabetes Care Management Program, Taiwan

	HbA1c-CV (%) standardized mean difference					
	Unmatched population (N= 27,257)			Matched population (N= 16,530)		
Variables	≤8.4% vs. 8.4%-17.1%	≤8.4% vs. >17.1%	8.4%-17.1% vs. >17.1%	≤8.4% vs. 8.4%-17.1%	≤8.4% vs. >17.1%	8.4%-17.1% vs. >17.1%
Sociodemographic factors						
Gender						
Female	0.01	0.08	0.07	-0.02	0.00	0.02
Male	-0.01	-0.08	-0.07	0.02	0.00	-0.02
Age (years)	0.01	0.12	0.11	-0.04	0.03	0.07
Lifestyle behaviors						
Smoking	-0.01	-0.10	-0.09	0.03	-0.02	-0.05
Alcohol drinking	-0.03	-0.06	-0.03	0.01	-0.01	-0.02
Diabetes-related variables						
Duration of diabetes (years)	0.01	0.18	0.17	-0.06	0.08	-0.13
Type of hypoglycemic drug use						
No medication	0.07	0.07	0.00	0.02	-0.04	-0.06
One oral hypoglycemic drug	0.15	0.18	0.03	0.03	-0.06	-0.09
Two oral hypoglycemic drugs	0.03	0.03	0.00	0.04	0.04	0.00
Three oral hypoglycemic drugs	-0.07	-0.07	-0.01	-0.02	0.02	0.04
>3 oral hypoglycemic drugs	-0.04	-0.08	-0.05	0.01	-0.01	-0.02
Insulin	-0.02	0.03	0.06	-0.04	-0.01	0.04
Insulin+ oral hypoglycemic drug	-0.13	-0.14	-0.01	-0.06	0.00	0.06
Drug-related variables						
Hypertension drug treatment	0.06	0.16	0.10	-0.03	0.02	0.04
Glucocorticoids	0.00	-0.01	-0.01	0.01	0.00	-0.01
Comorbidity						

Obesity	0.00	0.05	0.05	-0.02	0.00	0.02
CAD	-0.01	0.05	0.06	-0.03	0.02	0.05
CHF	-0.03	-0.02	0.01	-0.03	0.02	0.05
Cancer	0.00	-0.01	-0.01	0.02	0.00	-0.02
Hyperlipidemia	0.05	0.14	0.08	-0.02	0.01	0.03
Hypertension	0.02	0.14	0.12	-0.05	0.01	0.06
Atrial fibrillation	0.00	0.00	0.00	-0.02	0.00	0.02
Chronic hepatitis	-0.03	0.00	0.03	-0.03	0.01	0.04
Diabetic retinopathy	-0.02	-0.01	0.02	0.00	0.03	0.02
Hypoglycemia	0.00	0.00	0.00	0.01	0.01	-0.01
Pneumonia	-0.01	-0.02	-0.01	0.00	0.02	0.01
Other diseases of the upper respiratory tract	0.02	0.02	0.00	0.00	0.00	0.00
Acute respiratory infections	0.01	0.03	0.02	0.01	0.02	0.01
Blood biochemical measurement						
Fasting plasma glucose	-0.19	-0.38	-0.20	0.00	-0.02	-0.03
HbA1c	-0.24	-0.52	-0.28	0.02	-0.02	-0.04
FPG-CV	-0.23	-0.66	-0.46	0.03	-0.07	-0.10

CAD: coronary artery disease; CHF: congestive heart failure; FPG-CV: coefficient of variation of fasting plasma glucose; HbA1c-CV: coefficient of variation of HbA1c.
